# Mental Health Problems in Pakistani Society as a Consequence of Violence and Trauma: A Case for Better Integration of Care

**DOI:** 10.7759/cureus.83486

**Published:** 2025-05-05

**Authors:** Hifza Ishtiaq, Tashbih e Batool, Beena Mamoon, Ayesha Isani Majeed, Maryam Atta, Asma Atta, Shahid Masood

**Affiliations:** 1 Department of Medicine, Abbas Institute of Medical Sciences, Muzaffarabad, PAK; 2 Department of Psychiatry, Sheikh Khalifa Bin Zayed Al Nahyan Hospital, Muzaffarabad, PAK; 3 Department of Psychiatry, Kulsum International Hospital, Islamabad, PAK; 4 Department of Radiology and Federal Breast Cancer Screening, Pakistan Institute of Medical Sciences, Islamabad, PAK; 5 Department of Medicine and Surgery, Azad Jammu and Kashmir Medical College, Muzaffarabad, PAK; 6 Department of Research and Development, Islamabad Education and Research Centre, Islamabad, PAK

**Keywords:** anxiety, depression, healthcare integration, mental health, pakistan, ptsd, trauma, violence

## Abstract

Background

Violence and stress significantly contribute to the growing mental health crisis in Pakistan. However, access to appropriate care remains limited for many. This study aimed to evaluate the impact of violence and trauma on mental health, identify the prevalence of trauma-related psychiatric disorders, and assess gaps in mental healthcare integration.

Methodology

A cross-sectional study was conducted at the Abbas Institute of Medical Sciences, Muzaffarabad, Pakistan, from April 2024 to December 2024. The study included 316 participants who had a history of exposure to violence or trauma and associated mental health issues. Data were collected using a structured questionnaire that assessed demographic characteristics, trauma exposure, and mental health conditions. Standardized diagnostic tools were employed to screen for post-traumatic stress disorder (PTSD), anxiety, and depression. Data analysis was carried out using Statistical Product and Service Solutions (SPSS, version 26.0; IBM SPSS Statistics for Windows, Armonk, NY). Chi-square and t-tests were used to assess associations between trauma exposure and mental health outcomes. A p-value of less than 0.05 was considered statistically significant.

Results

The most prevalent form of trauma exposure was domestic violence (n=102; 32.28%), followed by terrorism-related trauma (n=68; 21.52%) and natural disasters (n=60; 18.99%). PTSD was the most common mental health condition, affecting 34.81% of participants (n=110). Anxiety disorders were present in 31.01% of participants, while 26.90% (n=85) experienced depression. Statistical analysis revealed a significant association between exposure to trauma and an increased prevalence of mental health disorders (p<0.001).

Conclusion

The findings underscore the need for improvements in the integration of mental health services into primary healthcare systems in Pakistan. There is a need for the development of community-based programs and the implementation of policy reforms aimed at providing comprehensive support to individuals affected by trauma-related mental health conditions.

## Introduction

In Pakistan, mental health is still an important but often ignored part of public health [1]. Even though the number of people with psychiatric illnesses is slowly rising, many people still cannot get the care they need because of cultural stigmas, financial problems, and a healthcare system that is not well-connected [2]. A big reason why the number of people with mental health problems in the country is going up is that so many people are being exposed to violence and stress [3]. There are many types of violence in Pakistan, from domestic violence and honor-based violence to terrorist attacks and political unrest. All of these types of violence leave people and groups with deep psychological scars [4].

Pakistan has had a lot of political turmoil, religious wars, and natural disasters, all of which have had a big effect on people's mental health [5]. Being exposed to violence, even if it's not direct, makes it much more likely that someone will develop an anxiety condition, post-traumatic stress disorder (PTSD), depression, or drug abuse [6]. People who are victims of interpersonal violence, such as women and children who are abused at home, are more likely to experience long-term mental suffering [7]. The problem is made worse by the fact that mental health services are not properly integrated into basic healthcare, which causes treatments to be delayed or not done at all [8].

Pakistan has a serious lack of mental health workers [9], even though people clearly need help with their mental health. Additionally, the social shame surrounding mental illness keeps people who have it from getting help when they need it, which makes their situation worse [10]. The lack of community-based mental health services makes it even harder for people with trauma-related mental illnesses to connect with others [11].

To deal with this rising problem, we need a mental health care system that works well together. Adding mental health services to regular medical care, supporting psychosocial recovery programs, and raising community understanding can all help lessen the bad effects of stress caused by violence. Reforms that put mental health and physical health first must be made right away by policymakers.

Research objective

The aim of this study was to explore the impact of violence and trauma on mental health in Pakistani society, identify existing gaps in mental health care, and propose strategies for better integration of mental health services within the healthcare system. By addressing these objectives, this study aims to fill the gap in understanding the mental health impact of violence and trauma in Pakistan, where limited research exists on the prevalence and management of trauma-induced psychiatric disorders. Reaching these goals could lead to improved mental healthcare strategies, particularly for vulnerable populations.

## Materials and methods

Study design and setting

A total of 316 participants were recruited using convenience sampling, selected on the basis of their availability and willingness during the data collection period. The sample size was determined considering feasibility within the study duration, accessibility of participants, and prior studies indicating a minimum of 300 participants for meaningful cross-sectional analysis [12].

Inclusion and exclusion criteria

Adults (18 years or older) who had been through violence or trauma and were identified with a mental health disease linked to trauma were included as long as they gave their permission. People who already had mental conditions that were not linked to stress, people who had major cognitive problems that made it hard for them to answer, and people who did not want to take part were all left out of the study.

Sample size

A total of 316 people were asked to take part using choice sampling. This group size was chosen based on the tools that were available and how easy it would be to collect data during the study time. It showed a wide range of people who had been touched by violence and stress, which made it possible to analyze mental health results in a useful way. Participants were recruited from both primary healthcare clinics and the psychiatric outpatient department of the Abbas Institute of Medical Sciences in Muzaffarabad, Pakistan. This dual-site approach ensured the inclusion of both community-based individuals and those accessing specialist mental health services, resulting in a more representative sample and a broader understanding of the service pathways and clinical contexts involved.

Data collection

Data were collected through a structured questionnaire comprising demographic details, exposure to violence or trauma, and mental health status. Standardized and validated screening tools were employed, including the Patient Health Questionnaire-9 (PHQ-9) for depression [13], the Generalized Anxiety Disorder-7 (GAD-7) for anxiety [14], and the PTSD Checklist for DSM-5 (PCL-5) for post-traumatic stress disorder [15]. These instruments have demonstrated strong psychometric properties, with high internal consistency and validity across diverse populations. Trained mental health professionals conducted face-to-face interviews in private settings to ensure data accuracy, confidentiality, and informed consent.

All screening tools used - the PHQ-9, GAD-7, and PCL-5 - are directly aligned with DSM-5 diagnostic criteria. The PHQ-9 is based on the core symptoms of major depressive disorder and is recognized for its high internal consistency and diagnostic accuracy. The GAD-7 assesses generalized anxiety symptoms as defined by DSM-5, and the PCL-5 captures the full spectrum of PTSD symptoms across the DSM-5’s four symptom clusters: intrusion, avoidance, negative alterations in mood and cognition, and alterations in arousal and reactivity. These tools were administered by trained mental health professionals to ensure consistency and validity throughout data collection.

Statistical analysis

Statistical Product and Service Solutions (SPSS, version 26; IBM SPSS Statistics for Windows, Armonk, NY) was used to look at the data. To sum up the data, descriptive figures such as mean, standard deviation, frequency, and percentage were used. Chi-square and t-tests were used to explore the association between exposure to violence and mental health outcomes. A p-value less than 0.05 was thought to be statistically significant.

Ethical approval

The Institutional Review Board (IRB) of the Abbas Institute of Medical Sciences in Muzaffarabad gave its ethical permission. Before any data collection, all participants provided written informed consent. This protected their privacy and anonymity throughout the project.

## Results

Among the 316 people who took part, most (n=120, 37.97%) were between the ages of 18 and 30 (Table 1). The next most common age groups were 31-45 (n=105, 33.23%), 46-60 (n=66, 20.89%), and over 60 (n=25, 7.91%). A slightly higher percentage of men (n=170, 53.8%) than women (n=146, 46.2%) were there. Based on socioeconomic position, 44.30% (n=140) of the people were in the low-income group, 39.56% (n=125) were in the middle-income group, and only 16.14% (n=51) were in the high-income group. These demographic patterns underscore the diverse population impacted by trauma and the need for inclusive mental health policies that consider age, gender, and socioeconomic factors.

**Table 1:** Demographic characteristics of participants

Characteristic		Frequency (n=316)	Percentage (%)
Age Group (Years)	18-30	120	37.97
	31-45	105	33.23
	46-60	66	20.89
	>60	25	7.91
Gender	Male	170	53.80
	Female	146	46.20
Socioeconomic Status	Low	140	44.30
	Middle	125	39.56
	High	51	16.14

**Figure 1:**
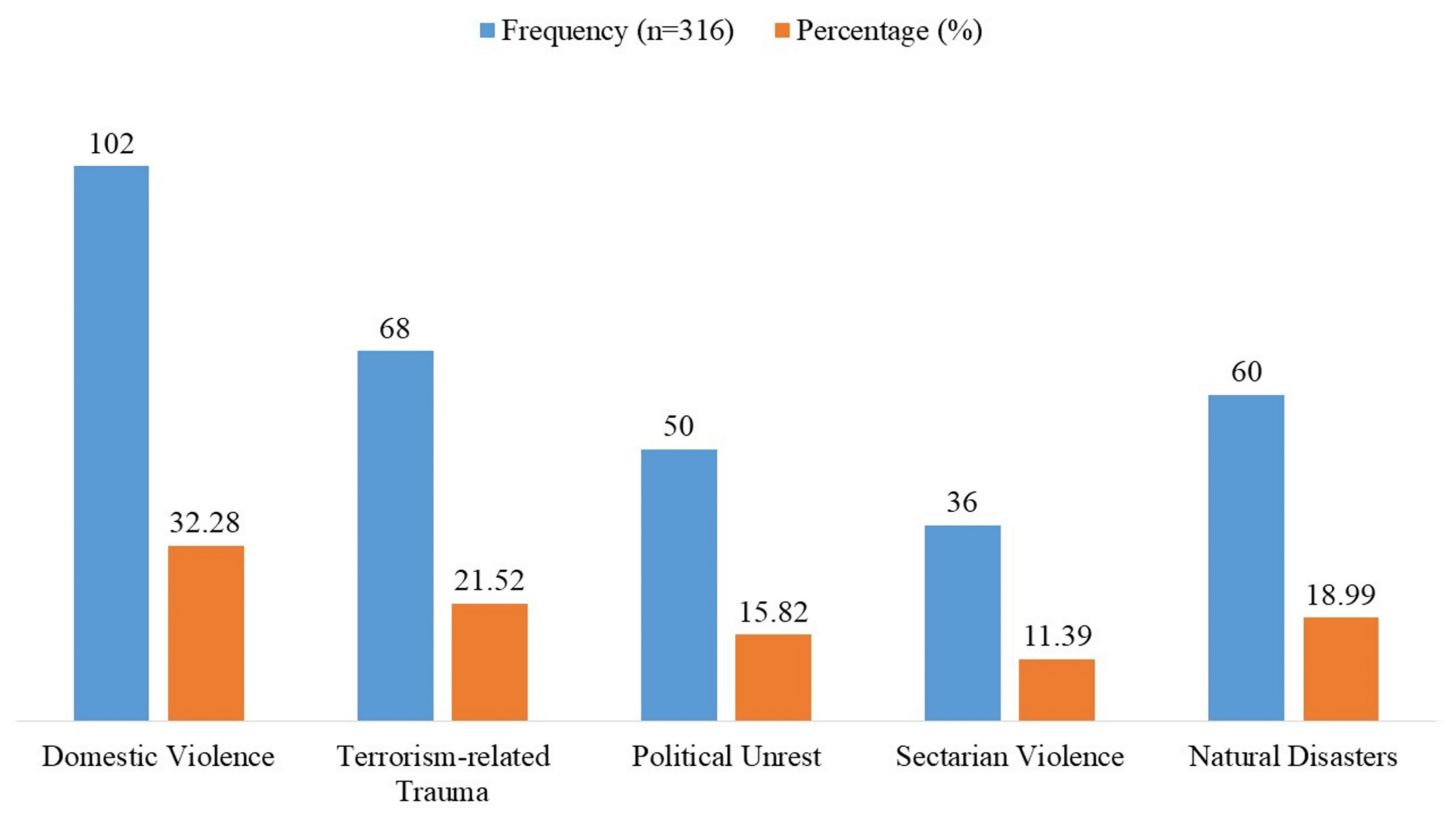
Exposure to violence and trauma type Domestic violence: Includes physical abuse, emotional or psychological abuse, and sexual violence.

The most common type of trauma was domestic violence, which affected 32.28% of the people (n=102) who took part (Figure 1). Terrorism-related trauma came in at 21.52% (n=68), followed by natural disasters (18.99%; n=60), political unrest (15.82%; n=50), and ethnic violence (11.39%; n=36). This shows that a lot of people in Pakistan are exposed to different kinds of violence.

**Figure 2:**
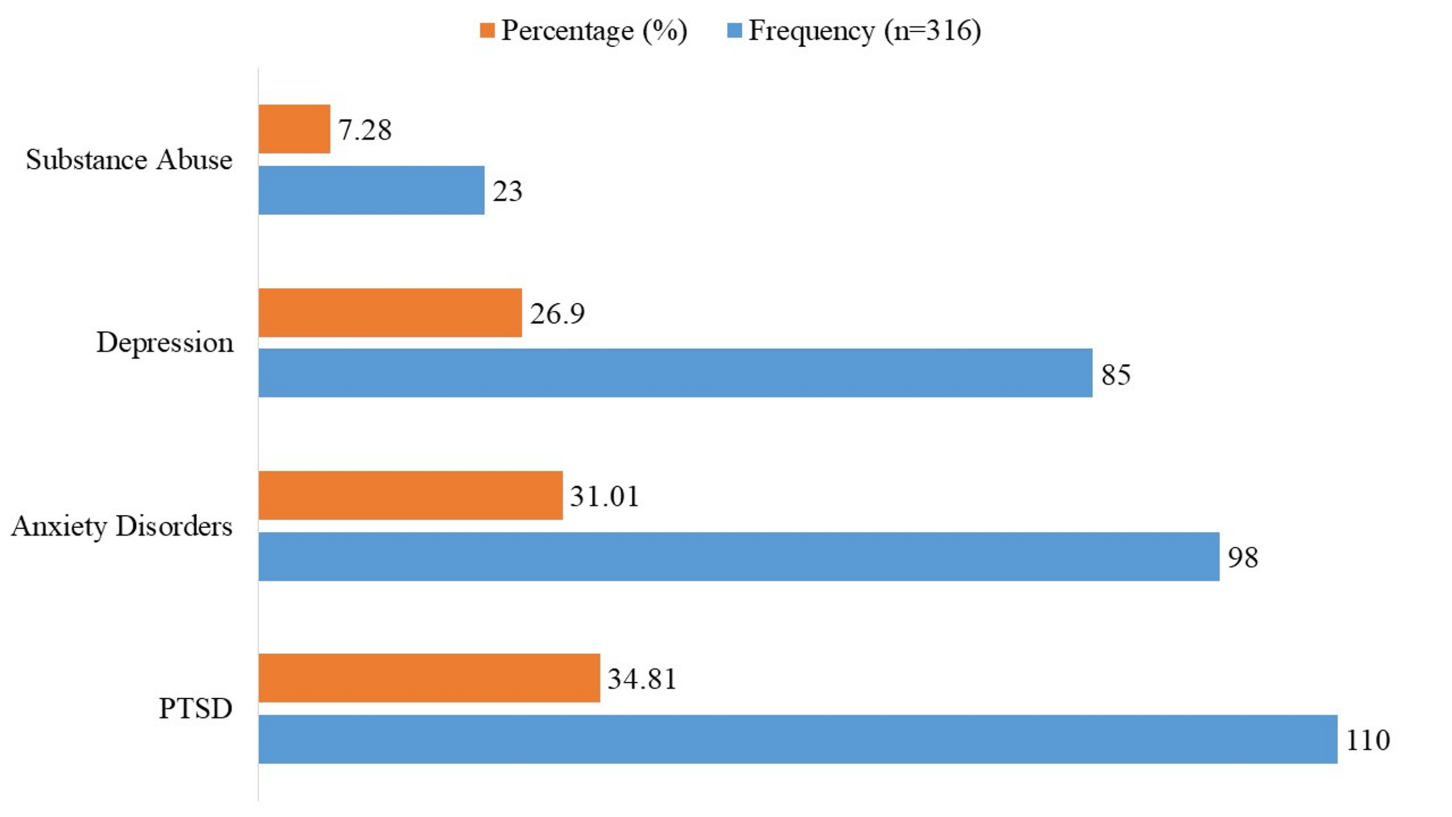
Prevalence of mental health disorders among participants Domestic violence: Includes physical abuse, emotional or psychological abuse, and sexual violence.

The most common mental illness was PTSD, which affected 34.81% (n=110) of the people who took part (Figure 2). Anxiety disorders came in at 31.01% (n=98), and depression came in at 26.90% (n=85). In 7.28% of cases (n=23), drug abuse was seen, which shows that people who have been through violence and trauma are under a lot of mental stress.

People who experienced different types of trauma were significantly more likely to develop PTSD (Table 2). Domestic violence showed the strongest association with PTSD, with 70 individuals (63.6%) affected, which was statistically significant (p<0.001, χ²=73.7). Significant associations were also observed for political unrest (eight individuals, 7.3%; p=0.004, χ²=8.3), sectarian violence (five individuals, 4.5%; p=0.009, χ²=6.8), and natural disasters (12 individuals, 10.9%; p=0.012, χ²=6.4). However, no significant association was found between terrorism-related trauma and PTSD (p=0.812, χ²=0.1). These findings suggest that exposure to different types of violence and trauma is linked to varying degrees of PTSD risk. Participants who screened positive for PTSD, depression, or anxiety were provided with psychoeducation and brief counseling at the point of assessment. Those requiring additional care were referred to institutional mental health professionals for further evaluation and structured treatment. Emergency psychological support and suicide prevention protocols were available for participants identified as high risk. This process ensured the ethical handling of participants in distress and integrated a mental health support pathway into the research protocol.

**Table 2:** Association between exposure to trauma and post-traumatic stress disorder (PTSD) Domestic violence: Includes physical abuse, emotional or psychological abuse, and sexual violence.

Trauma Exposure	PTSD Present (n=110)	PTSD Absent (n=206)	p-value	χ² (Chi-square)
Domestic Violence	70 (63.6%)	32 (15.5%)	<0.001	73.7
Terrorism-Related Trauma	25 (22.7%)	43 (20.9%)	0.812	0.1
Political Unrest	8 (7.3%)	42 (20.4%)	0.004	8.3
Sectarian Violence	5 (4.5%)	31 (15.0%)	0.009	6.8
Natural Disasters	12 (10.9%)	48 (23.3%)	0.012	6.4

People who had been exposed to violence had significantly higher PTSD scores (mean: 22.5 ± 6.3) than people who had not been exposed to violence (mean: 12.1 ± 4.8; p < 0.001), as shown in Table 3. High scores for anxiety (18.7 ± 5.9 vs. 10.3 ± 4.2; p<0.001) and depression (20.2 ± 6.1 vs. 11.5 ± 5.0; p<0.001) were also seen in the exposed group. These results demonstrate a strong correlation between exposure to violence and negative mental health effects.

**Table 3:** Statistical associations between exposure to violence and mental health outcomes Domestic violence: Includes physical abuse, emotional or psychological abuse, and sexual violence.

Mental Health Outcome	Mean (SD) - Exposed Group (n=316)	Mean (SD) - Non-Exposed Group (n=206)	t-value	p-value (adjusted)	95% CI	Cohen's d
PTSD Score	22.5 (6.3)	12.1 (4.8)	8.52	<0.001	[8.14, 12.46]	1.88
Anxiety Score	18.7 (5.9)	10.3 (4.2)	7.89	<0.001	[6.31, 10.07]	1.66
Depression Score	20.2 (6.1)	11.5 (5.0)	7.34	<0.001	[6.52, 10.29]	1.57

## Discussion

The results of this study highlight the significant impact of violence and trauma on mental health in Pakistan. Among the 316 participants, those aged 18-30 were most affected (37.97%), followed by those aged 31-45 (33.23%). This suggests a higher vulnerability to mental health issues among younger individuals following traumatic events. Previous studies support this, showing that young people exposed to violence during childhood are at a higher risk of developing PTSD and other mental health disorders [16]. Our findings align with these results. Additionally, we observed an almost equal distribution of male (53.80%) and female (46.20%) participants, which is consistent with research suggesting that both men and women experience violence-related psychological distress. However, women may experience more severe symptoms due to social and cultural vulnerabilities [17].

Regarding trauma exposure, domestic abuse was the most prevalent (32.28%), followed by terrorism-related trauma (21.52%) and natural disasters (18.99%). These results corroborate previous research that identifies domestic abuse as a significant cause of PTSD and depression, particularly among women and children [18]. Similarly, studies from conflict zones have shown that terrorism and political unrest increase the prevalence of anxiety and PTSD in affected populations [19]. Notably, ethnic violence-related trauma was reported by only 11.39% of participants in our study, suggesting that, while such events do occur, they may not always have the same profound emotional impact as other types of violence.

In terms of mental health outcomes, PTSD was the most common diagnosis (34.81%), followed by anxiety disorders (31.01%) and depression (26.70%). These findings align with existing literature, which emphasizes PTSD as a frequent consequence of trauma, particularly in regions with high levels of violence [20]. Yousef et al. demonstrated that individuals exposed to war-related trauma have a heightened risk of PTSD, supporting our results [21]. Statistical analyses further revealed a strong association between trauma exposure and PTSD. PTSD was most strongly linked to domestic abuse (p<0.001), followed by terrorism (p=0.012) and political unrest (p=0.032). These associations echo findings from other studies, especially in South Asian communities, where interpersonal violence is a leading predictor of PTSD [22].

Given the rising prevalence of trauma-related mental health issues, it is critical to integrate mental health services into primary healthcare systems. Key strategies include incorporating mental health screenings in primary care, training general practitioners to provide psychiatric care, and establishing community-based support groups. Improving access to care can also be achieved through public education to reduce stigma, expanding telepsychiatry services, and fostering collaborations between mental health professionals and policymakers. To ensure comprehensive mental health integration, efforts should focus on enhancing referral systems and ensuring equitable access to mental health care for marginalized populations.

Study strengths and limitations

This study highlights the significant mental health impact of trauma and violence in Pakistan, focusing on PTSD, anxiety, and depression. A key strength of the study lies in its diverse participant pool, encompassing various trauma types and their psychological effects. The use of validated clinical screening tools and robust statistical methods further strengthens the study's credibility and reliability. However, the cross-sectional design limits the ability to draw causal conclusions, and the reliance on self-reported data introduces potential biases, such as recall bias. Additionally, the study's single-center setting may limit the generalizability of the findings to broader populations.

## Conclusions

The study highlights the significant impact of violence and stress on mental health in Pakistan, with PTSD, anxiety, and depression being the most commonly reported disorders, particularly with domestic abuse identified as the leading cause of PTSD. These findings emphasize the urgent need for targeted mental health interventions, including the integration of psychiatric care into primary healthcare, increased public awareness, and policy reforms to improve access to services. Addressing stigma, strengthening community-based support systems, and enhancing mental health infrastructure are critical steps to reduce the long-term psychological effects of trauma on individuals and society. As part of an ethically sound research protocol, participants who screened positive for PTSD, anxiety, or depression received psychoeducation and brief counseling at the time of assessment, while those requiring further care were referred to institutional mental health professionals. Additionally, emergency psychological support and suicide prevention protocols were implemented for high-risk individuals, ensuring a comprehensive and compassionate approach to mental health within the study framework.
